# The Label-Free Fluorescence Detection of Inorganic and Organic Mercury Based on DNA-Templated Gold Nanoclusters

**DOI:** 10.3390/bios16040218

**Published:** 2026-04-14

**Authors:** Zhiqiang Chen, Kangyao Zhang

**Affiliations:** 1 School of Food Engineering, Zhangzhou Institute of Technology, Zhangzhou 363000, China; 2 School of Advanced Manufacturing, Fuzhou University, Quanzhou 362251, China; zhangky@fzu.edu.cn

**Keywords:** inorganic mercury, organic mercury, gold nanoclusters, ssDNA aptamer, fluorescence detection

## Abstract

Heavy metal mercury is one of the most significant and toxic environmental contaminants. Its inorganic form (Hg^2+^) and organic form (organic mercury, OrHg) can cause irreversible harm to human health and the ecological environment, and the latter is particularly prone to bioaccumulation and bioamplification in the food chain. Therefore, there is an urgent need for a rapid, reliable and specific detection of Hg^2+^ and OrHg to evaluate the potential risk for human health. Here, a novel label-free fluorescent sensing platform based on ssDNA aptamer (A_A-T7_)-templated AuNCs was established for sensitive recognition and specific detection of Hg^2+^ and OrHg. In the presence of OrHg, the fluorescence of pure A_A-T7_-templated AuNCs was visibly enhanced through forming Ag/AuNCs based on Ag^0^-doped AIEE effect. However, they were obviously quenched because of generating non-fluorescent Au/Ag/Hg ANPs via metallophilic interactions among Au^3+^, Ag^+^, and Hg^2+^ (5d^10^-4d^10^-5d^10^) when only Hg^2+^ existed. This fluorescent sensing platform could detect as low as 20.0 nM (4.0 ng Hg/g) and has a good linear detection range, with target concentrations ranging from 0.25 μM to 2.00 μM, recoveries of 98.0–108.0%, and RSD ≤ 5.0%. Low-toxic A_A-T7_-templated AuNCs could be used for cytotoxicity analysis and intracellular fluorescent imaging. The method has been successfully applied to the determination of Hg^2+^ and OrHg in tap water, seawater and dried golden pomfret fish muscle samples, demonstrating promising prospects for the assay of mercury species in environmental samples and aquatic products to ensure human health and food safety.

## 1. Introduction

Heavy metals play a fundamental role in industry, serving as key materials that underpin economic and social development [1]. Along with the rapid progress in the global industrial economy over the past decades, however, a series of unprecedentedly solemn environmental problems of heavy metals has been emerging in an endless stream [2]. According to relevant data, up to 8000 tons of mercury are released into the environment each year by natural processes and human activities [3,4]. Among them, coal combustion and small-scale artisanal gold mining are the main sources of mercury released by human activities. As one of the most significant and toxic heavy metal sources, mercury is ranked by the World Health Organization (WHO) as the world’s third-largest pollutant and is also listed as a global contaminant by the United Nations Environment Programme (UNEP) [5].

Different mercury species exhibit different biological toxicities. For example, elemental mercury (Hg^0^) vapor released mostly from burning fossil fuels to the atmosphere is oxidized to soluble inorganic mercury ion (Hg^2+^), diffusing into the soil and aquatic environments where bacteria translate Hg^2+^ into much higher toxic organic mercury (OrHg) represented by methylmercury (MeHg) and ethylmercury (EtHg) via methylation [6,7,8]. They were primarily accumulated in predatory fishes and delivered to human bodies through the food chain of the ecological system, as well as eventually bioamplification in the human brain, triggering irreversible diseases, teratogenic effects, genetic mutations, and even death, especially under the circumstance of long-term intake of organic mercury-rich fishes [9,10,11,12,13]. It can thus be seen that OrHg has stronger biological toxicity than Hg^2+^ and other mercury species.

Therefore, many countries and international institutions have imposed strict restrictions on the use of mercury-containing products and on mercury levels in drinking water, and have also issued advisories regarding the weekly tolerable intake of fish with higher OrHg content [14,15,16,17,18,19,20,21,22]. Among these restrictive measures, China and the European Union (EU) have revised and limited the maximum MeHg content in most carnivorous fish and their products to as low as 1.0 mg/kg. However, the most fundamental or challenging task at present remains the monitoring and determination of OrHg concentration in the aquatic environment and in seafood using technological means to prevent mercury poisoning and ensure food safety.

The detection techniques for mercury species have progressed from single-instrument to combined-instrument, and ultimately to a portable, rapid assay system. Conventional methods, which integrate separation techniques including capillary electrophoresis (CE), gas chromatography (GC) and high-performance liquid chromatography (HPLC) with detection techniques like inductively coupled plasma mass spectrometry (ICP-MS), atomic fluorescence spectrometry, and atomic absorption spectroscopy (AAS), exhibit high precision in the determination of Hg^2+^ [23,24,25,26,27,28,29,30]. However, they suffer from limitations, including high cost, low sensitivity and selectivity, reliance on complex operations and specialized techniques, and lengthy analysis times, which cannot meet the requirements for on-site detection. Emerging methods, such as hybrid techniques combining surface-enhanced Raman spectroscopy (SERS) and ion-imprinted polymers (IIPs), have merely enhanced the sensitivity and selectivity for Hg^2+^ [31,32,33].

With the interdisciplinary application of materials in the chem-biological field, colorimetric methods that utilize biomolecular element recognition based on DNA-based sensors and functional nanomaterials have been developed in recent years to enable simple, real-time detection of Hg^2+^ or MeHg [34,35,36,37]. Even more surprisingly, subsequent fluorescence methods overcome the shortcomings of colorimetric methods, such as matrix interference, poor specificity and low sensitivity and detection limits. Unfortunately, fluorescence methods are primarily based on organic fluorescent dyes that cause environmental pollution, have short fluorescence lifetimes and are unable to detect MeHg and EtHg simultaneously [38,39]. Lately, fluorescent noble metal nanoclusters (MNCs), with nanoscale substances consisted of pure noble metal atoms (or their alloys), include gold nanoclusters (AuNCs) as well as silver nanoclusters (AgNCs), and they are regarded as a promising alternative because of their strong fluorescence emission, unique physicochemical properties, high sensitivity and specificity, good water compatibility and easy to modification [40,41,42,43,44]. In addition, low-cost and relatively stable deoxyribonucleic acid (DNA) sequences with a thymine-rich (T-rich) base can bind specifically to Hg^2+^ and are often used to build biosensors for detecting Hg^2+^ [45]. Inspired by the above methods, herein we propose a novel biosensor for rapidly determining OrHg that combines self-assembled AuNCs and T-rich single-stranded DNA (ssDNA) to form DNA-templated AuNCs, where T-rich ssDNA and DNA-templated AuNCs serve as recognition and signal elements, respectively. This study not only expands the innovative applications of AuNCs for mercury species detection but also provides a simple and reliable method for rapid detection of Hg^2+^ and OrHg in water and seafish samples, thereby providing technical support for improving the safety of drinking water and seafood consumption.

## 2. Materials and Methods

### 2.1. Chemical Reagents

All the ssDNA sequences (Table S1 in Supplementary Materials) we used in this experiment were synthesized and verified through HPLC purification and mass spectrometry analysis by Sangon Biotech Co., Ltd. (Shanghai, China). Methyl-mercuric chloride (MeHgCl) and ethyl-mercuric chloride (EtHgCl) were purchased from Dr. Ehrenstorfer GmbH (Augsburg, Germany). Chloroauric acid trihydrate (HAuCl_4_·3H_2_O) and mercury nitrate monohydrate (Hg(NO_3_)_2_·H_2_O) were produced by J&K Scientific Group (Beijing, China). Silver nitrate (AgNO_3_), hydroxylamine hydrochloride (HONH_3_Cl) and citric acid (CA) were obtained from Sigma-Aldrich Company (Shanghai, China). L(+)-ascorbic acid (AA) and sodium borohydride (NaBH_4_) were purchased from Sinopharm Chemical Reagent Co., Ltd. (Shanghai, China). Nitric acid (HNO_3_), sodium dihydrogen phosphate dihydrate (NaH_2_PO_4_·2H_2_O), disodium hydrogen phosphate dihydrate (Na_2_HPO_4_·2H_2_O) and disodium ethylenediaminetetraacetate dihydrate (Na_2_EDTA·2H_2_O) were obtained from Xilong Chemical Co., Ltd. (Guangzhou, China). Phosphate-buffered saline (PBS) solution was received from Hyclone (Logan, UT, USA). Tris-(hydroxymethyl) aminomethane (Tris) was obtained from Merck (Darmstadt, Germany). RPMI1640 cell culture medium and fetal bovine serum (FBS) were purchased from Gibco (Logan, UT, USA). The cell viability was assessed using a Cell Counting Kit-8 (CCK-8) from Dojindo (Kumamoto-ken, Japan).

Before the formal experiment, the concentration of each ssDNA sequence was also accurately quantified by the optical density (OD) at a wavelength of 260 nm (OD_260_) based on its individual absorption coefficients. Phosphate buffer (PB) solution (10 mM, pH 7.5) was prepared by mixing equivalent Na_2_HPO_4_ and NaH_2_PO_4_ solutions (10 mM), and its pH was adjusted with 10 mM Na_2_HPO_4_ solution. In this work, all reagents were at least grade of analytical reagent (AR) and experimental water was generated from a sterile Millipore Milli-Q ultrapure water system (18.2 MΩ·cm).

### 2.2. Main Instruments

The fluorescence spectroscopy and UV–vis absorption spectroscopy were obtained by Tecan’s Infinite M200 PRO multifunctional microplate reader (Männedorf, Switzerland). The confocal fluorescence imaging analysis was performed by Nikon A1R HD25 confocal laser scanning microscopy (Tokyo, Japan). Transmission electron microscope (TEM) images and energy-dispersive X-ray (EDX) spectra analysis were measured by FEI Tecnai G2 F20 U-TWIN (Portland, OR, USA). The concentrations of MeHg and EtHg were confirmed using an Agilent 7800 ICP-MS (Santa Clara, CA, USA). The pH was measured by the Ohaus Starter 2100/E acidometer (Ohaus Corporation, Parsippany, NJ, USA). The particle size was analyzed using dynamic light scattering (DLS) by Malvern Panalytical Zetasizer Nano ZS90 analyzer (Malvern, UK). The single-channel/multichannel pipette and low-temperature high-speed centrifuge were purchased from Eppendorf AG (Hamburg, Germany).

### 2.3. Experimental Methods

#### 2.3.1. Preparation of DNA-Templated AuNCs

DNA-templated AuNCs were prepared by using the A_A-T7_ aptamer (5’-AAA AAA AAA AAA AAA AAA AAA AAA AAA AAA GTT CTT TGT TAA AAA TTC TTT GTT CGG-3’) as template, referring to the reported methods previously [46,47]. Briefly, 60 μL of 5 mM HAuCl_4_, 20 μL of 15 μM A_A-T7_ aptamer, 20 µL of freshly prepared 0.6 mM NaBH_4_ and 150 µL of 10 mM Tris-HNO_3_ buffer solution (pH 7.5) were mixed gently in a new 1.5 mL non-enzyme Eppendorf (EP) centrifuge tube. The entire mixed solution was kept away from light at 95 °C for 10 min to form A_A-T7_-templated AuNCs, which would be stored in the dark at 4 °C for subsequent experiments.

#### 2.3.2. Specific Fluorescent Detection for Hg^2+^ and OrHg

For inorganic and organic mercury detection, 180 µL of the as-prepared A_A-T7_-templated AuNCs solution was subsequently added to a new 1.5 mL non-enzyme EP tube. Before shaking gently, 10 µL of 0.16 mM Ag^+^ solution and 10 µL of Hg^2+^ or OrHg at different concentrations were sequentially added. Then 20 µL of 0.6 mM NaBH_4_ was added to the aforementioned mixture, which was incubated at 25 °C for 5 min. Eventually, the excitation wavelength was set at 350 nm, and the fluorescence emission spectra of the final reaction system solution were scanned from 450 to 650 nm. The concentration of Hg^2+^ or OrHg was calculated in accordance with the rate of change in the fluorescence intensity (ΔF/F_0_), where ΔF = |F − F_0_|, F_0_ and F represented the fluorescence intensity of A_A-T7_-templated AuNCs at 514 nm in the absence and presence of Hg^2+^ or OrHg, respectively.

#### 2.3.3. Pretreatment and Determination of Actual Samples

Tap water and seawater samples collected from the lab tap and coastal waters of Zhangzhou, Fujian, China, were directly filtered through a 0.22 µm membrane filter to remove suspended particles and macromolecules. After that, the filtrate could be directly analyzed for Hg^2+^ content in tap water or seawater using the above procedure. In order to determine OrHg in tap water or seawater, however, before being analyzed by the method proposed in Section 2.3.2, the above filtrate needs to be further treated by AA for 1 h to remove Hg^2+^ and nitrogen (N_2_) for 5 min to remove Hg^0^ according to a previously reported method [48].

The selected seafish samples were dried golden pomfret fish muscle collected from coastal waters off Zhangzhou, Fujian, China. The dried golden pomfret fish muscle was extracted, concentrated to near-dryness, and finally diluted with 5.0 mL of Tris-HNO_3_ buffer solution (pH 7.5) using the method described in reference [49]. At last, the Hg^2+^ and OrHg in golden pomfret fish muscle were separately detected with the same method in Section 2.3.2, respectively. Tap water, seawater and seafish samples spiked with different concentrations of Hg^2+^ or OrHg were prepared and analyzed in the same manner to determine recovery (Rec.).

#### 2.3.4. Assessment of Cell Viability and Intracellular Fluorescent Imaging Analysis

Human cervical cancer cells (HeLa cells) were initially cultured in RPMI 1640 medium containing 10% heated, inactivated FBS. HeLa cells were washed twice with PBS solution and digested with 0.25% Trypsin-0.53 mM EDTA (TE) solution. Then 200 μL of the above A_A-T7_-templated AuNCs system solution with/without 1.0 μM Hg^2+^ or OrHg was added into RPMI 1640 cell culture medium and co-incubated for 6 h at 37 °C with 5% CO_2_. Cell viability assessment and intracellular fluorescent imaging analysis were performed as described below.

To assess cell viability, after adding 10 μL of CCK-8 solution, HeLa cells were incubated for 4 h in the dark. The absorbance at 450 nm (OD_450_) of the final solution was measured, where cell viability (%) = (OD_450_ of mercury species group—OD_450_ of blank control group)/(OD_450_ of negative control group—OD_450_ of blank control group) × 100%. Furthermore, HeLa cells were treated with the A_A-T7_-templated AuNCs system solution with/without Hg^2+^ or OrHg, and incubated in laser confocal culture dishes for 6 h. After being washed twice with PBS, HeLa cells were stained for intracellular fluorescent imaging analysis with an excitation wavelength of 488 nm. Moreover, a fluorescent emission filter (band-pass filter) is used in Nikon A1R HD25 confocal laser scanning microscopy for fluorescent imaging analysis, and the fluorescent emission range is collected from 400 nm to 750 nm.

## 3. Results and Discussion

### 3.1. The Principle of Specific Fluorescent Detection for Hg^2+^ and OrHg

DNA is a fascinating template and desirable option for synthesizing fluorescent MNCs, such as AuNCs, with potential applications in biosensing. Due to the strong affinity of adenine (A) for binding with Au^3+^ to form the A-Au^3+^-A complex, especially, adenine-rich (A-rich) ssDNA can toughly template with AuNCs to self-assembly ssDNA-templated AuNCs that have higher fluorescence quantum yield, more stable fluorescence response and longer fluorescence lifetime than AgNCs [50,51]. The binding affinity of ssDNA with transition metal ions may be influenced by the orientation of ssDNA. Meanwhile, thymine-rich (T-rich) ssDNA display different binding affinity with transition metal ions following the order of OrHg, Hg^2+^, Ag^+^ and Au^3+^, so T-rich ssDNA can be employed as a scaffold for specific recognition and binding with target mercury species [38,52]. In addition, the incorporation of Ag^+^ improves material stability and significantly enhances the fluorescence intensity of AuNCs via “Ag effect”, which may be because the electronic contribution of Ag^0^ within the alloy structure promotes radiative recombination in aggregated states and thereby heightens the fluorescence response [53].

**Scheme 1:**
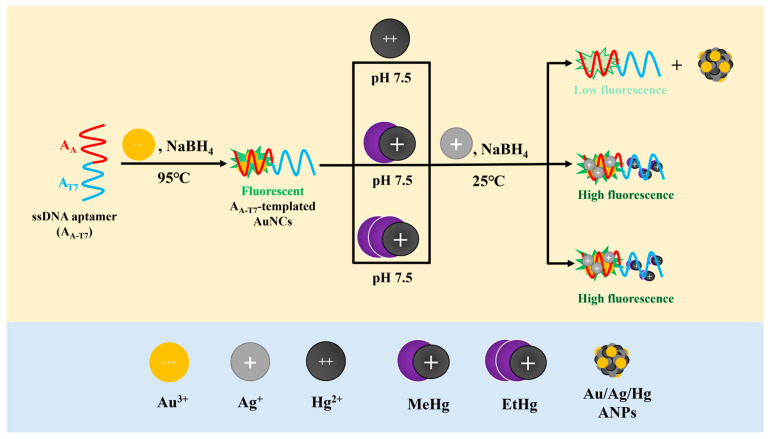
Experimental principle of specific fluorescent detection for inorganic and organic mercury based on A_A-T7_-templated AuNCs.

Inspired by the above research, we here built a novel label-free fluorescent sensing platform based on ssDNA aptamer (A_A-T7_)-templated AuNCs for specific detection of Hg^2+^ and OrHg. A-rich A_A_ and T-rich A_T7_ were the two parts that made up A_A-T7_. The former primarily formed fluorescent A_A_-templated AuNCs, while the latter specifically bound to OrHg. As shown in Scheme 1, A_A-T7_-templated AuNCs were generated by the binding of bases A in A_A-T7_ with Au^3+^ to form an A-Au^3+^ complex, where Au^3+^ was then reduced to Au^0^ by NaBH_4_, and eventually Au^0^ was assembled along the contour of the A_A-T7_ template to shape fluorescent AuNCs [54]. When Hg^2+^ or OrHg was introduced in the presence of Ag^+^, the fluorescence intensity of A_A-T7_-templated AuNCs would be significantly changed. More specifically, if there was Hg^2+^ in the final solution of the target system, Hg^2+^ was combined with Au^3+^ and Ag^+^, and reduced by NaBH_4_ to generate non-fluorescent Au/Ag/Hg alloy nanoparticles (ANPs) via metallophilic interactions among Au^3+^, Ag^+^, and Hg^2+^ (5d^10^-4d^10^-5d^10^) [46], so the fluorescence of A_A-T7_-templated AuNCs could be quenched. Conversely, due to stronger binding affinity than Hg^2+^, OrHg was bound preferentially on A_T7_, but free Ag^+^ in solution was reduced by NaBH_4_ to Ag^0^ gathering around AuNCs, namely forming Ag/AuNCs. This ultimately led to fluorescence enhancement of A_A-T7_-templated AuNCs via an Ag^0^-doped aggregation-induced emission (AIE) effect.

### 3.2. Characterization of Label-Free A_A-T7_-Templated AuNCs-Based Fluorescent Sensing Platform

**Figure 1:**
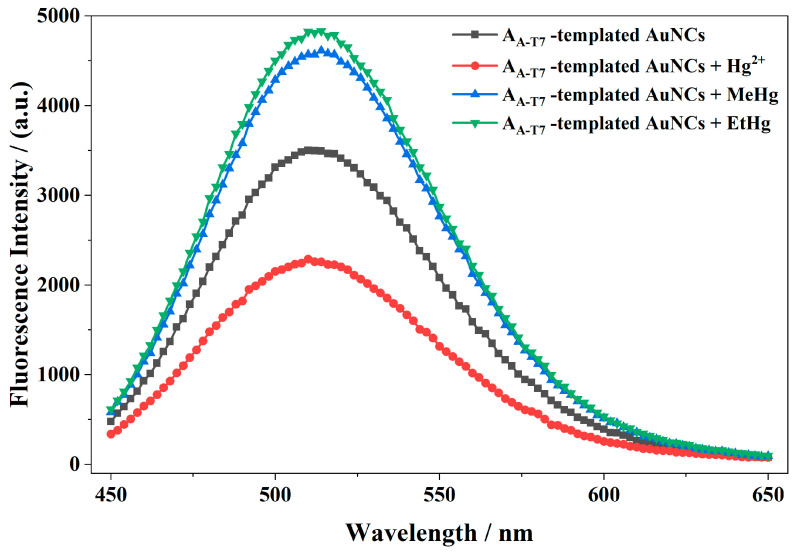
The fluorescence emission spectra of A_A-T7_-templated AuNCs with/without Hg^2+^ or OrHg.

To verify the effectiveness and feasibility of the label-free A_A-T7_-templated AuNCs-based fluorescent sensing platform we proposed, the fluorescence emission spectra of the A_A-T7_-templated AuNCs with/without Hg^2+^ or OrHg were first measured using the same procedures shown in Scheme 1. At 350 nm excitation wavelength, as illustrated in Figure 1, the pure A_A-T7_-templated AuNCs showed an obvious fluorescent response in the wavelength range from 450 nm to 650 nm, and exhibited a maximum emission peak around 514 nm, which demonstrated the successful synthesis of A_A-T7_-templated AuNCs. Moreover, their fluorescence quantum yield (QY) was expected to approach 6%, consistent with the results reported in the literature [50,55]. While Hg^2+^ was introduced, the fluorescence intensity of the system’s final solution was significantly quenched; on the contrary, the presence of OrHg sensibly enhanced the fluorescence intensity of the system’s final solution. This phenomenon indicated the difference between Hg^2+^ and OrHg in affecting the fluorescence intensity of A_A-T7_-templated AuNCs and also preliminarily confirmed our deduction.

**Figure 2:**
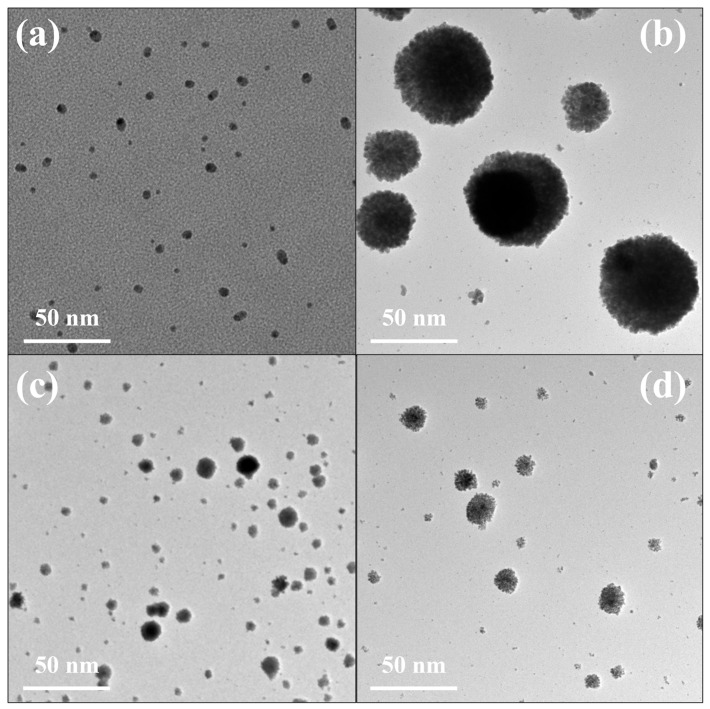
TEM images of the A_A-T7_-templated AuNCs without any mercury species (**a**), as well as with Hg^2+^ (**b**), MeHg (**c**) or EtHg (**d**).

To further support our inference, the morphological characterization of A_A-T7_-templated AuNCs with/without mercury species was performed using TEM images (Figure 2). Found from Figure 2a, there were quasi-spherical A_A-T7_-templated AuNCs with an average diameter (AD) ~5 nm, as well as good dispersibility and stability. When Hg^2+^ existed in the final solution, however, the AD was increased to ~50 nm (Figure 2b) and key elements, including Au, Ag and Hg, were determined by EDX spectra for elemental analysis among these particles (Figure S1b), suggesting that Au/Ag/Hg ANPs were formed by reducing mixed Au^3+^, Ag^+^ and Hg^2+^. In the presence of OrHg, the mean size of particles lay between 5 nm and 50 nm (Figure 2c,d). What’s more, the corresponding EDX spectra (Figure S1c,d) only displayed the signal elemental signatures of Au and Ag, but not Hg. This possible reason was due to multi-particle aggregation or the Ag^0^-doped AIEE effect, but not to any chemical interaction, because the formed A_A-T7_-templated AuNCs displayed the same absorption spectra in the presence or absence of mercury species (Figure S2). Ag^+^ was reduced to Ag^0^ and aggregated on the surface of A_A-T7_-templated AuNCs (especially in the A_A_ section of A_A-T7_) to form Ag/AuNCs, and meanwhile, OrHg was bound preferentially on the A_T7_ section of A_A-T7_. Furthermore, the surface coverage of the AuNCs with A_A-T7_ was determined by the reported method to verify that there was a difference in the quantification of A_A-T7_ attached on AuNCs in the presence or absence of mercury species, following the order of OrHg > Hg^2+^ (Figure S3), consistent with the order of binding affinity [38,56]. Additionally, the particle-size distribution obtained by DLS analysis of A_A-T7_-templated AuNCs under different conditions was analyzed, further verifying the above deduction (Figure S4).

### 3.3. Optimization of ssDNA Aptamer

**Figure 3:**
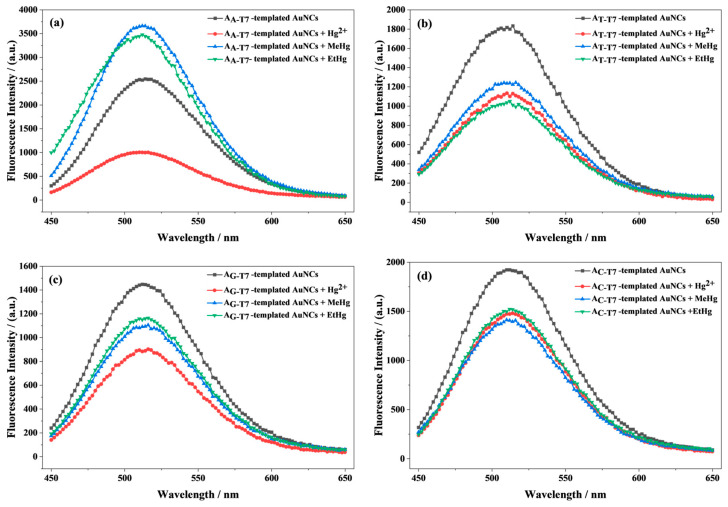
Optimization of ssDNA aptamer for specific detection of Hg^2+^ and OrHg. (**a**) A_A-T7_; (**b**) A_T-T7_; (**c**) A_G-T7_; (**d**) A_C-T7_.

It was widely acknowledged that DNA had four bases, including adenine (A), thymine (T), guanine (G) and cytosine (C). As an interesting template molecule with secondary structure, ssDNA was suitable for templating MNCs and had specific recognition of mercury species [51,57,58]. According to the literature, A-rich ssDNA could be used for the synthesis of ssDNA-templated AuNCs by A-Au^3+^-A base pairs, but T-rich ssDNA tended to bind with Hg^2+^ instead of OrHg to form T-Hg^2+^-T structures [38,46,59]. Based on the above facts, we designed the novel ssDNA aptamer, consisting of two segments: segment 1 (T-rich ssDNA, A_T7_) and segment 2 (any base-rich ssDNA, A_rich-in-base_). Under the above same procedures as shown in Scheme 1, to ascertain the optimal A_rich-in-base_ of ssDNA aptamer, four different types of sequence rich in A, T, G and C bases (namely A_A_, A_T_, A_G_ and A_C_) were set to explore the fluorescence characteristics. As shown in Figure 3, the fluorescence intensity of the system solution was relatively low and readily quenched by Hg^2+^ and OrHg, regardless of the selected ssDNA aptamer (A_T-T7_, A_G-T7,_ or A_C-T7)_. A possible reason was that, owing to their insensitivity to Au^3+^, the ssDNA-templated AuNCs were unstable and vulnerable to Hg^2+^ and OrHg attack, thereby affecting their fluorescence emission [60]. In other words, only when A_A-T7_ was used in this reaction system, the synthesized AuNCs exhibited a strong fluorescence signal at 514 nm, which could also be quenched and enhanced by Hg^2+^ and OrHg, respectively. A_A-T7_ was therefore identified as the optimal ssDNA aptamer for specificity analysis of Hg^2+^ and OrHg.

### 3.4. Optimization of Other Important Experimental Conditions

In this study, many factors had an influence on assay results. Under the above optimal experimental conditions, in addition to the discussed ssDNA aptamer (A_A-T7_), other important experimental conditions, such as the concentration of A_A-T7_, the reducing agent and its concentration, the pH of the Tris-HNO_3_ buffer solution, and the concentration of Au^3+,^ should be considered to improve analytical performance.

As mentioned above, A_A-T7_ was the most suitable scaffold for establishing a stable AuNC fluorescent system, so we first evaluated A_A-T7_ at concentrations ranging from 2.0 μM to 20 μM using the same procedures and optimal conditions. Figure S5 shows the fluorescence emission spectra obtained with our proposed method. With increasing concentrations of A_A-T7_, the fluorescence intensity of the system solution in the presence of different mercury species was gradually enhanced. However, when concentrations of A_A-T7_ were increased between 2.0 μM and 10 μM, it was difficult to judge Hg^2+^ and OrHg since their closely or nearly overlapping fluorescence emission spectra could not be distinctly distinguished, indicating that a low concentration of A_A-T7_ was hard to form sufficient templated AuNCs without significant fluorescence quenching or enhancement effect during the subsequent process. At the concentration of 20 μM, nevertheless, the fluorescence intensity of A_A-T7-te_mplated AuNCs was quenched by Hg^2+^ and OrHg, which implied possibly forming plenties of Au/Ag/Hg ANPs. Specific fluorescence detection by Hg^2+^ and OrHg met our expectations only at 15 μM. Hence, we considered 15 μM to be the optimal concentration of A_A-T7_.

The role of the reducing agent was to reduce Au^3+^, Ag^+^ and Hg^2+^ to form self-assembled AuNCs or ANPs, so its reduction ability was closely related to the quality of the analysis results. AA, CA, NaBH_4_ and HONH_3_Cl were used and compared here. Under the above same procedures and optimal conditions, as shown in Figure S6, AA, CA and HONH_3_Cl did not trigger an obvious fluorescence phenomenon, probably because of their relatively low reducibility and stability compared with NaBH_4_ [61]. By utilizing NaBH_4_ as a reducing agent, an evident fluorescence signal and notable reaction distinction occurred between Hg^2+^ and OrHg groups. That is, NaBH_4_ was the optimal reducing agent, serving both reducing and stabilizing roles. Furthermore, we selected 0.6 mM as the optimal concentration of NaBH_4_ after optimization (Figure S7).

The pH directly influenced the size and fluorescence emission characteristics of A_A-T7_-templated AuNCs [62]. Between pH 6.5 and pH 8.0, four candidate pH values were set, and the result was displayed in Figure S8, under the same procedures and optimal conditions as above. We note that when the pH value of the analysis system was pH ≤ 7.0, not only was the fluorescence intensity of A_A-T7_-templated AuNCs relatively low, but also Hg^2+^ or OrHg could not clearly influence the fluorescence response of A_A-T7_-templated AuNCs, demonstrating that fluorescent A_A-T7_-templated AuNCs were suppressed and possessed similar binding affinity to Hg^2+^ and OrHg, under weakly acidic or neutral conditions. However, excessively high pH value (pH 8.0) decreased the fluorescence intensity of A_A-T7_-templated AuNCs, which was also significantly quenched by Hg^2+^ and OrHg. Therefore, pH 7.5 was chosen as the most suitable pH as a result of specific fluorescence quenching by Hg^2+^ and fluorescence enhancement by OrHg at this pH value.

Au^3+^ was adsorbed and locally enriched through electrostatic and coordination interactions with the A_A-T7_ backbone to form AuNCs by reduction [63]. As a key precursor for synthesizing AuNCs, Au^3+^ had a decisive influence on nucleation, growth and optical properties of A_A-T7_-templated AuNCs. Figure S9 shows the fluorescence emission spectra of the system solution at different Au3+ concentrations, obtained under the same procedures and optimal conditions. The results indicated that excessively high concentrations of Au^3+^ might readily cause untemplated growth or transform into large-sized nanoparticle structures [55], but they did not offer an adequate amount of Au^3+^ for generating fluorescent A_A-T7-te_mplated AuNCs in the presence of low concentrations of Au^3+^. On the contrary, 5.0 mM of Au^3+^ resulted in A_A-T7_-templated AuNCs exhibiting higher fluorescence intensity and specifically detecting Hg^2+^ and OrHg. Accordingly, 5.0 mM was confirmed as the optimal concentration of Au^3+^ and used in further procedures.

### 3.5. The Selectivity of the Developed Method for Hg^2+^ and OrHg Detection

**Figure 4:**
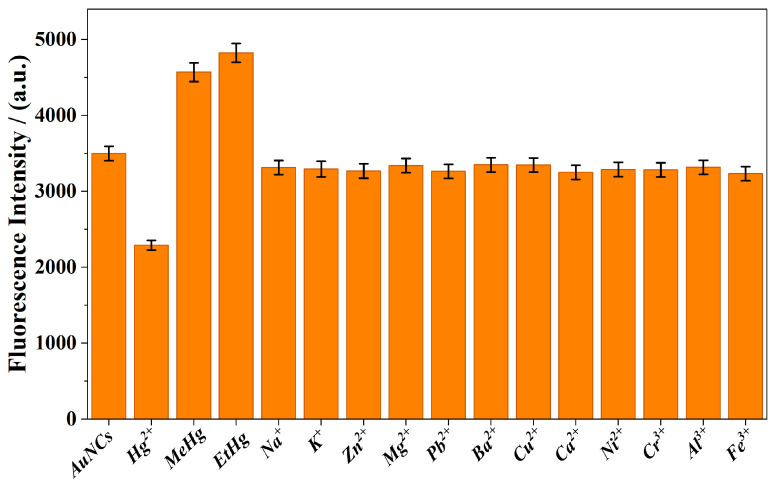
The selectivity of the developed method for Hg^2+^ and OrHg detection. The fluorescence intensity of the assay system at 514 nm was recorded in the presence of common coexisting metallic ions with A_A-T7_-templated AuNCs under the above optimal conditions.

To evaluate the specificity of our fluorescent sensing platform, potential interference effects of common coexisting metallic ions, including K^+^, Na^+^, Mg^2+^, Zn^2+^, Pb^2+^, Ba^2+^, Ca^2+^, Ni^2+^, Cu^2+^, Fe^3+^, Cr^3+^ and Al^3+^ should be taken into consideration by comparing their differences in fluorescence response under the above optimal experimental conditions. Under the above same procedures and optimal conditions, the concentration of common coexisting metallic ions (100 μM) was 100 times higher than that of mercury species. The fluorescence emission spectra (Figure 4) showed that these common coexisting metallic ions had no significant impact on fluorescence intensity, indicating that they did not interfere with the assay of Hg^2+^ and OrHg. This further illustrated that, in coexisting natural environments, our method showed strong specificity and selectivity for simultaneous detection of Hg^2+^ and OrHg.

### 3.6. The Analytical Performance of Our Proposed Method

**Figure 5:**
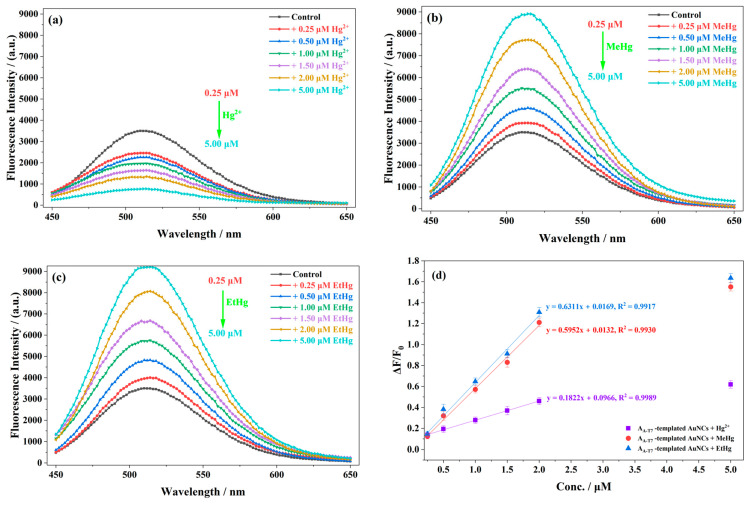
The analytical performance of A_A-T7_-templated AuNCs-based fluorescent sensing platform. (**a**) The fluorescence intensity of the assay system in the presence of A_A-T7_ templated AuNCs with different Conc. of Hg^2+^; (**b**) The fluorescence intensity of the assay system in the presence of A_A-T7_ templated AuNCs with different Conc. of MeHg; (**c**) The fluorescence intensity of the assay system in the presence of A_A-T7_ templated AuNCs with different Conc. of EtHg; (**d**) The relationship between the ΔF/F_0_ and Conc. of Hg^2+^, MeHg or EtHg ranging from 0.25 μM to 5.00 μM and their calibration curves.

To achieve the optimal analytical performance of proposed method, the fluorescence emission spectra from 450 to 650 nm with different concentrations of Hg^2+^ and OrHg were collected under the above same procedures and optimal conditions, selected above and the results displayed in Figure 5 indicated a continuous decrease or increase in fluorescence intensity at 514 nm with the increase concentration of Hg^2+^ or OrHg from 0.25 μM to 5.00 μM. Linear equations also revealed good linear relationship between rate of change in the fluorescence intensity (ΔF/F_0_) and their concentration (Conc.) ranging from 0.25 μM to 2.00 μM, and their equations of the regression curve: ∆F/F_0_ = 0.1822 × C + 0.0966 (R^2^ = 0.9989) for Hg^2+^; ∆F/F_0_ = 0.5952 × C + 0.0132 (R^2^ = 0.9930) for MeHg; ∆F/F_0_ = 0.6311 × C + 0.0169 (R^2^ = 0.9917) for EtHg, where C representing the concentration of Hg^2+^ or OrHg with the unit of μM (Figure 5d). It was worth noting that the sensitivity for determining MeHg was virtually the same as that for EtHg, so it could be regarded as detecting the concentration of OrHg. In addition, the theoretical limit of detection (3σ/S) was calculated to be 20.0 nM (4.00 ng Hg/g) and 25.0 nM (5.00 ng Hg/g) for Hg^2+^ and OrHg, respectively. These values are well below the maximum mercury level (0.5 mg/kg) for food according to the latest Chinese National Food Safety Standard (GB 2762–2025). Therefore, we conclude that the analytical performance of our established label-free A_A-T7_-templated AuNCs-based fluorescent sensing platform was superior to that of the majority of reported methods for mercury assay [47,48,63,64,65,66,67] (Table 1).

### 3.7. Determination of Hg^2+^ and OrHg in Actual Samples

To ensure the reliability and practical applicability of the analysis results obtained with this fluorescent sensing method, actual samples were pretreated and analyzed for mercury species content under the same procedures and optimal conditions. Tap water sourced from a lab tap, seawater and seafish (dried golden pomfret fish muscle) samples collected from the coastal water of Zhangzhou, Fujian, China, were selected. Before the fluorescence assay, partially pretreated actual samples were spiked with different Conc. of Hg^2+^ or OrHg. As listed in Table 2 and Table 3, the content of Hg^2+^ or OrHg in tap water, seawater and dried golden pomfret fish muscle samples was accurately determined with Rec. of 98.0–108.0% and a relative standard deviation (RSD, *n* = 6) ≤ 5.0%. The above-obtained actual samples were also verified by capillary electrophoresis combined with ICP-MS (CE-ICP-MS) [49], yielding results similar to those obtained by CE-ICP-MS. The above facts indicate that the label-free fluorescent sensing method could be applied to specific detection and trace analysis of mercury species in actual samples.

### 3.8. A_A-T7_-Templated AuNCs for Cell Viability Analysis and Intracellular Fluorescent Imaging

To assess the application of A_A-T7_-templated AuNCs at the cellular level, their cytotoxicity and biocompatibility were eventually tested by a CCK-8 cell viability assay. The results (Figure S10) demonstrated that AA-T7-templated AuNCs were slightly to negligibly toxic and, conversely, that 1.0 μM of Hg^2+^ or OrHg was highly toxic. In addition, HeLa cells were incubated with A_A-T7_-templated AuNCs for intracellular confocal fluorescence imaging analysis. As shown in Figure S11, upon excitation at 488 nm, red fluorescent A_A-T7_-templated AuNCs were expressed in HeLa cells, indicating good cell imaging performance. Meanwhile, the introduction of trace Hg^2+^ or OrHg could effectively induce fluorescence quenching or enhancement, demonstrating that A_A-T7_-templated AuNCs were capable of intracellularly detecting trace mercury species in live cells.

## 4. Conclusions

A novel label-free fluorescent sensing platform based on ssDNA aptamer (A_A-T7_)-templated AuNCs was herein developed for specific detection of Hg^2+^ and OrHg. Due to stronger binding affinity to OrHg than Hg^2+^, the fluorescent A_A-T7_-templated AuNCs were enhanced visibly through forming Ag/AuNCs based on Ag^0^-doped AIEE effect, but they were obviously quenched as a result of generating non-fluorescent Au/Ag/Hg ANPs via metallophilic interactions among Au^3+^, Ag^+^, and Hg^2+^ (5d^10^-4d^10^-5d^10^). The proposed fluorescent sensing platform possessed a low limit of detection (20.0 nM for Hg^2+^ and 25.0 nM for OrHg) and a good linear relationship between ΔF/F_0_ and their concentration, ranging from 0.25 μM to 2.0 μM. This method has been successfully applied to the assay of Hg^2+^ and OrHg in tap water, seawater and dried golden pomfret fish muscle samples and is expected to promote the development of novel aptamer-based biosensors for the detection of mercury species in environmental samples and aquatic products to ensure human health and food safety.

Admittedly, though the proposed method has been mentioned above, there are still some potential drawbacks and limitations that need to be improved or explored further throughout the entire process. For example, the quantification and orientation of A_A-T7_ attached to AuNCs, as well as the binding affinity between A_A-T7_ and Hg^2+^ or OrHg, should be optimized and discussed in depth. Then, X-ray photoelectron spectroscopy (XPS) should be considered another effective approach to verify the actual state of mercury in the reaction system under four conditions. Furthermore, the current method detection limit is not sufficiently low and should be further reduced by optimizing additional influencing factors, such as the system’s temperature and the concentration of the Tris-HNO_3_ buffer solution. Additionally, in the field of evaluating the application of A_A-T7_-templated AuNCs at the cell level, several details such as cellular quantitative capability and uptake efficiency, as well as intracellular localization of A_A-T7_-templated AuNCs with/without Hg^2+^ or OrHg, should also be implemented and refined to fully elaborate the good analytical performance of this method.

## Figures and Tables

**Table 1:** Comparison of the analytical performance of the proposed method with other methods.

Methods	Mercury Species	Analytical Performance			References
		LOD (μM)	LOQ (nM)	Detection Range (μM)	
Fluorescent	OrHg	0.05	5.0	0.05–5.0	[47]
Colorimetric	Hg^2+^; OrHg	2.0–10.0	2.0–10.0	0.0–100.0	[48]
Fluorescent	Hg^2+^	0.5	12.0	0.5–2.5	[64]
Fluorescent	Hg^2+^	0.13	1.2	0.13–1.5	[40]
Fluorescent	Hg^2+^	0.1	83.0	0.1–100.0	[65]
Colorimetric	Hg^2+^; OrHg	0.01–0.026	2.6–30.0	0.01–1.5	[66]
Electrochemical	Hg^2+^	0.01	0.01	1.0 × 10^−6^–0.01	[67]
Fluorescent	Hg^2+^; OrHg	0.25	20.0–25.0	0.25–5.0	This work

**Table 2:** Analytical results of Hg^2+^ in tap water, seawater and dried golden pomfret fish muscle samples.

Samples	Added Hg^2+^ (μM)		Detected by Our Method			Verified by CE-ICP-MS (μM)
			Conc. (μM)	Rec. (%)	RSD (%)	
Tap water	1	0.00	-	-	-	-
	2	0.50	0.47	98.0	4.2	0.49
	3	1.00	1.02	102.0	3.6	1.01
	4	2.00	1.98	98.5	4.0	2.02
Seawater	1	0.00	-	-	-	-
	2	0.50	0.48	98.2	4.5	0.50
	3	1.00	1.14	104.6	3.2	1.02
	4	2.00	2.12	104.5	3.8	2.01
Fish muscle	1	0.00	0.08	-	5.0	0.07
	2	0.50	0.61	106.0	3.6	0.61
	3	1.00	1.07	99.0	2.9	1.08
	4	2.00	2.10	105.9	4.6	2.09

**Table 3:** Analytical results of OrHg in tap water, seawater and dried golden pomfret fish muscle samples.

Samples		Added OrHg (μM)		Detected by Our Method			Verified by CE-ICP-MS (μM)	
		MeHg	EtHg	Conc. (μM)	Rec. (%)	RSD (%)	MeHg	EtHg
Tap water	1	0.00	0.00	-	-	-	-	-
	2	1.00	0.00	1.02	104.9	3.0	1.02	0.0
	3	0.00	1.00	0.99	99.3	4.2	0.00	1.01
	4	0.50	0.50	0.98	99.0	4.5	0.51	0.49
Seawater	1	0.00	0.00	-	-	-	-	-
	2	1.00	0.00	0.99	99.4	4.2	1.01	0.00
	3	0.00	1.00	1.03	105.0	2.8	0.00	1.00
	4	0.50	0.50	1.01	102.6	3.2	0.50	0.51
Fish muscle	1	0.00	0.00	0.28	-	5.0	0.27	0.00
	2	1.00	0.00	1.25	98.3	4.6	1.25	0.01
	3	0.00	1.00	1.27	99.5	4.1	0.28	0.99
	4	0.50	0.50	1.29	108.0	2.6	0.76	0.52

## Data Availability

The details of the data supporting the reported results are available in the article or the Supplementary Materials. Further inquiries can be directed to the corresponding author.

## Supplementary Materials

The following supporting information can be downloaded at: https://www.mdpi.com/article/10.3390/bios16040218/s1, Figure S1: EDX spectra of A_A-T7_-templated AuNCs without any mercury species (a), as well as with Hg^2+^ (b), MeHg (c) or EtHg (d); Figure S2: The ultraviolet-visible absorption spectra of A_A-T7_-templated AuNCs without any mercury species, as well as with Hg^2+^, MeHg or EtHg; Figure S3: The surface coverage of the AuNCs with A_A-T7_ in the presence or absence of mercury species; Figure S4: Particle-size distribution from DLS analysis of A_A-T7_-templated AuNCs without any mercury species (a), as well as with Hg^2+^ (b), MeHg (c) or EtHg (d); Figure S5: Optimization of the concentration of A_A-T7_ ranging from 2.0 μM to 20 μM. (a) 2.0 μM; (b) 10 μM; (c) 15 μM; (d) 20 μM; Figure S6: Optimization of reducing agent. (a) AA; (b) CA; (c) NaBH_4_; (d) HONH_3_Cl; Figure S7: Optimization of the concentration of NaBH_4_ ranging from 0.2 mM to 0.8 mM. (a) 0.2 mM; (b) 0.4 mM; (c) 0.6 mM; (d) 0.8 mM; Figure S8: Optimization of pH value in system solution ranging from pH 6.5 to pH 8.0. (a) pH 6.5; (b) pH 7.0; (c) pH 7.5; (d) pH 8.0; Figure S9: Optimization of concentrations of Au^3+^ ranging from 3.0 mM to 6.0 mM. (a) 3.0 mM; (b) 4.0 mM; (c) 5.0 mM; (d) 6.0 mM; Figure S10: Cell viability analysis of A_A-T7_-templated AuNCs with/without Hg^2+^, MeHg or EtHg (*n* = 4); Figure S11: Intracellular confocal fluorescence imaging analysis. (a) Hela cells at bright field; (b) Hela cells at dark field; (c) Hela cells incubated with A_A-T7_-templated AuNCs at dark field; (d) Hela cells co-incubated with A_A-T7_-templated AuNCs and Hg^2+^ at dark field; (e) Hela cells co-incubated with A_A-T7_-templated AuNCs and MeHg at dark field; (f) Hela cells co-incubated with A_A-T7_-templated AuNCs and EtHg at dark field; Table S1: The sequences of synthesized ssDNA aptamer used in this work.

## Abbreviations

The following abbreviations are used in this manuscript:

WHO	World Health Organization
UNEP	United Nations Environment Programme
OrHg	Organic mercury
MeHg	Methylmercury
EtHg	Ethylmercury
EU	European Union
CE	Capillary electrophoresis
GC	Gas chromatography
HPLC	High-performance liquid chromatography
ICP-MS	Inductively coupled plasma-mass spectrometry
AFS	Atomic fluorescence spectrometry
AAS	Atomic absorption spectroscopy
SERS	Surface-enhanced Raman spectroscopy
IIPs	Ion-imprinted polymers
MNCs	Metal nanoclusters
AgNCs	Silver nanoclusters
AuNCs	Gold nanoclusters
ANPs	Alloy nanoparticles
DNA	Deoxyribonucleic acid
T-rich	Thymine-rich
A-rich	Adenine-rich
ssDNA	Single-stranded DNA
OD	Optical density
PBS	Phosphate buffered saline
PB	Phosphate buffer
FBS	Fetal bovine serum
CCK-8	Cell counting kit-8
AR	Analytical reagent
TEM	Transmission electron microscope
EDX	Energy dispersive X-ray
AD	Average diameter
DLS	Dynamic light scattering
EP	Eppendorf
TE	Trypsin-EDTA
AIEE	Aggregation-induced emission enhancement
QY	Quantum yield
Conc.	Concentrations
Rec.	Recoveries
RSD	Relative standard deviation
