# Supplementary material for: The Label-Free Fluorescence Detection of Inorganic and Organic Mercury Based on DNA-Templated Gold Nanoclusters
Source: Biosensors (Basel). 2026 Apr 14;16(4):218. doi: 10.3390/bios16040218 (PMC13115007; doi:10.3390/bios16040218)

biosensors

Supplementary data for:

The label-free fluorescence detection of inorganic and organic mercury based on DNA-templated gold nanoclusters

Zhiqiang Chen [1,*], Kangyao Zhang [2]

[1] School of Food Engineering, Zhangzhou Institute of Technology, Zhangzhou 363000, China; chenzhiqiang@fjzzit.edu.cn
[2] School of Advanced Manufacturing, Fuzhou University, Quanzhou 362251, China; zhangky@fzu.edu.cn
* Correspondence: chenzhiqiang@fjzzit.edu.cn

Table S1. The sequences of synthesized ssDNA aptamers used in this work.

Aptamer	Sequences (5′ → 3′)
$A_{A\text{-}T7}$	AAA AAA AAA AAA AAA AAA AAA AAA AAA AAA GTT CTT TGT TAA AAA TTC TTT GTT CGG
$A_{T\text{-}T7}$	GTT CTT TGT TAA AAA TTC TTT GTT CGT TCT TTG TTA AAA ATT CTT TGT TCG G
$A_{G\text{-}T7}$	GGG GGG AGG GGG GAG GGG GGA GGG GGG AGG GGG GGT TCT TTG TTA AAA ATT CTT TGT TCG G
$A_{C\text{-}T7}$	ACC CGA ACC TGG GCT ACC ACC CTT AAT CCC CGT TCT TTG TTA AAA ATT CTT TGT TCG G

Figure S1. EDX spectra of $A_{A\text{-}T7}$-templated AuNCs without any mercury species (a), as well as with Hg^{2+} (b), MeHg (c) or EtHg (d).

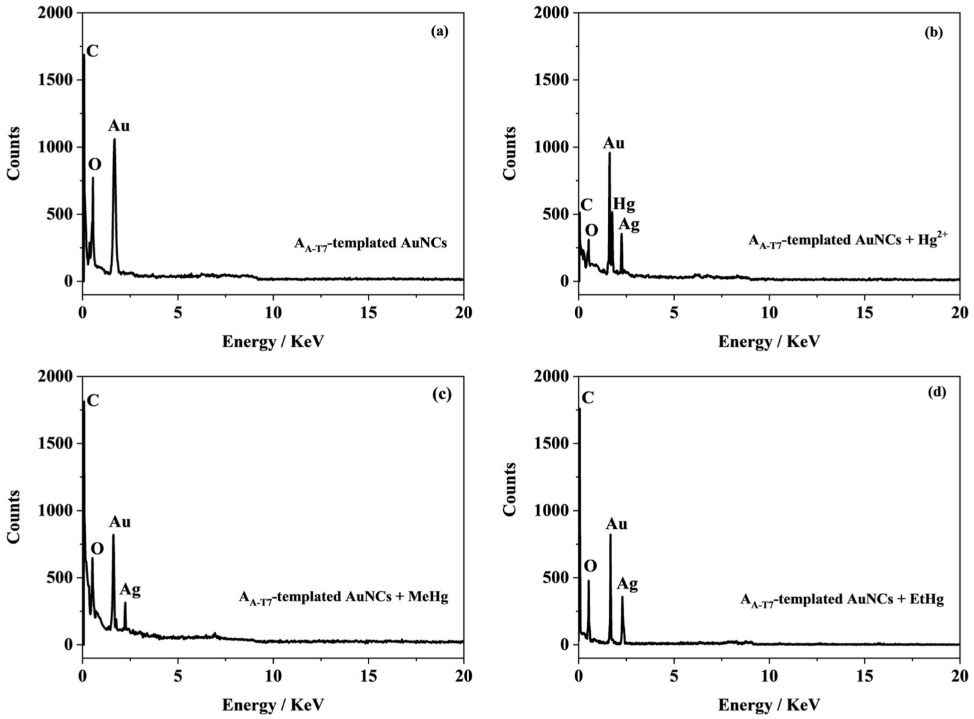

biosensors

Figure S2. The ultraviolet-visible absorption spectra of $A_{A\text{-}T7}$-templated AuNCs without any mercury species, as well as with Hg^{2+}, MeHg or EtHg.

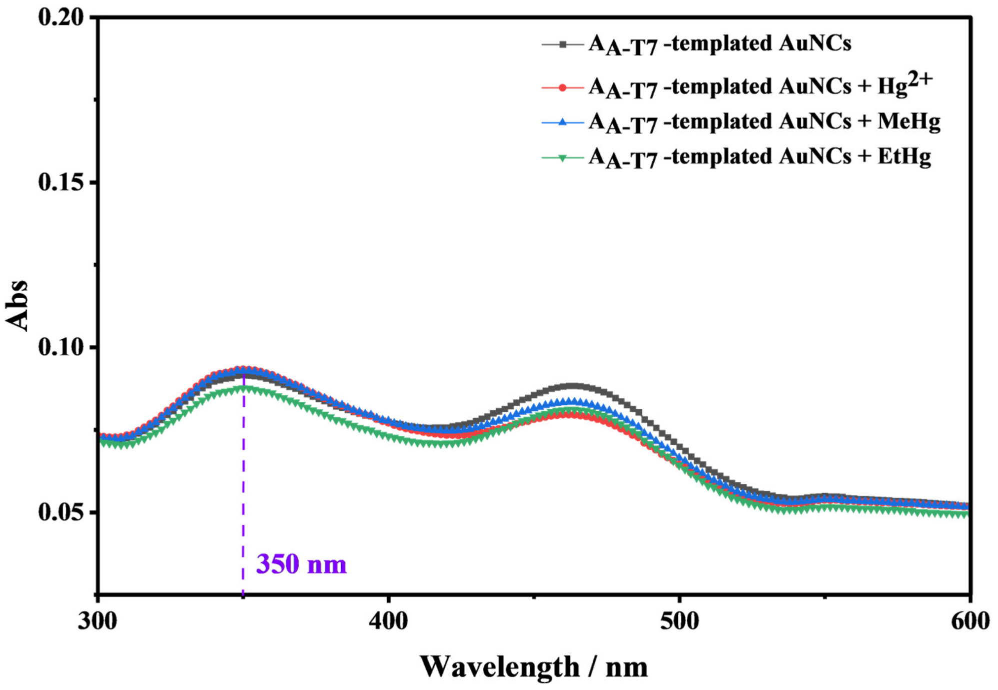

Figure S3. The surface coverage of the AuNCs with $A_{A\text{-}T7}$ in the presence or absence of mercury species.

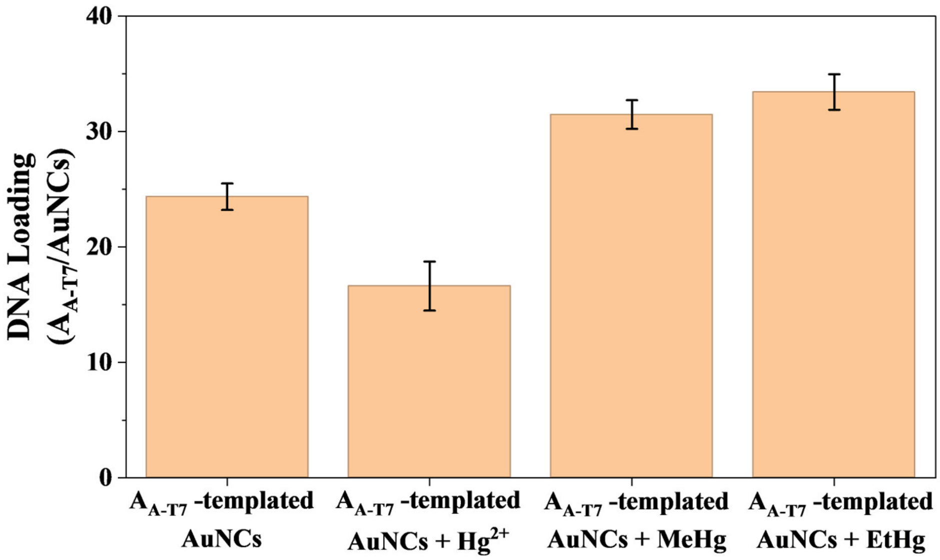

Figure S4. Particle-size distribution from DLS analysis of $A_{A\text{-}T7}$-templated AuNCs without any mercury species (a), as well as with Hg^{2+} (b), MeHg (c) or EtHg (d).

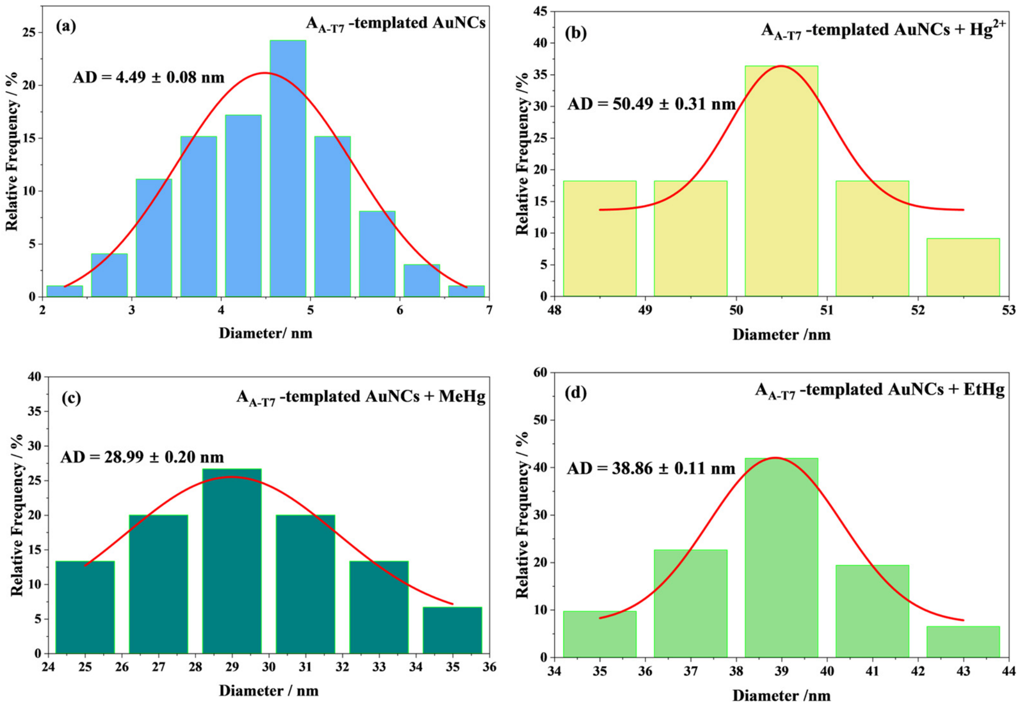

Figure S5. Optimization of the concentration of $A_{A\text{-}T7}$, ranging from 2.0 μM to 20 μM. (a) 2.0 μM; (b) 10 μM; (c) 15 μM; (d) 20 μM.

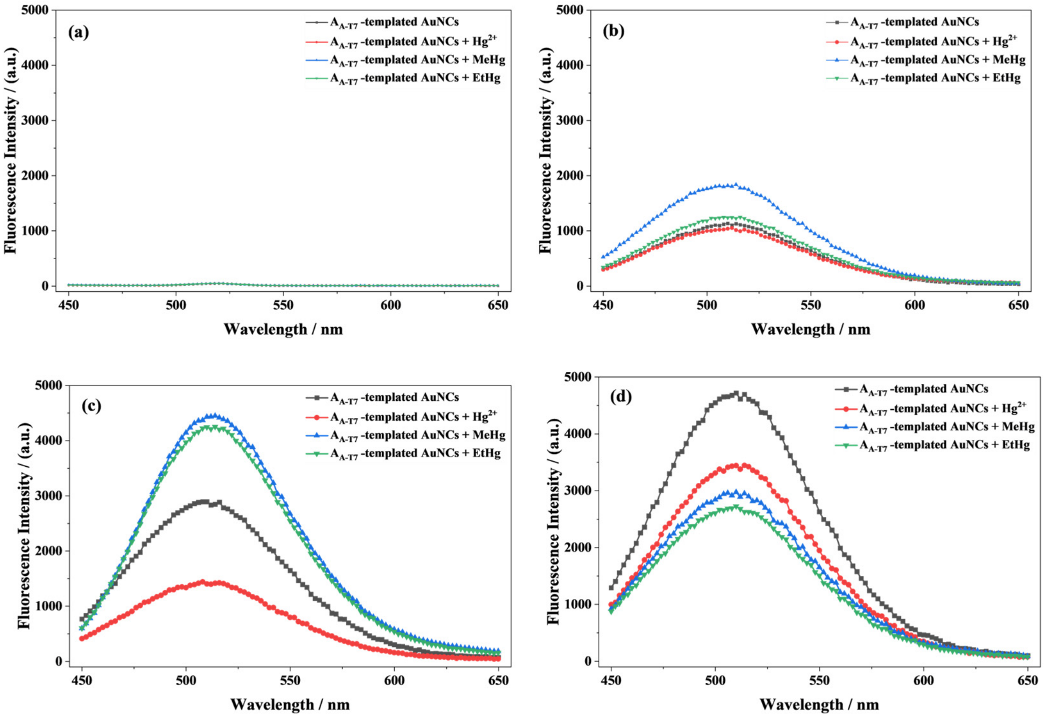

biosensors

Figure S6. Optimization of the reducing agent. (a) AA; (b) CA; (c) $NaBH_4$; (d) $HONH_3Cl$.

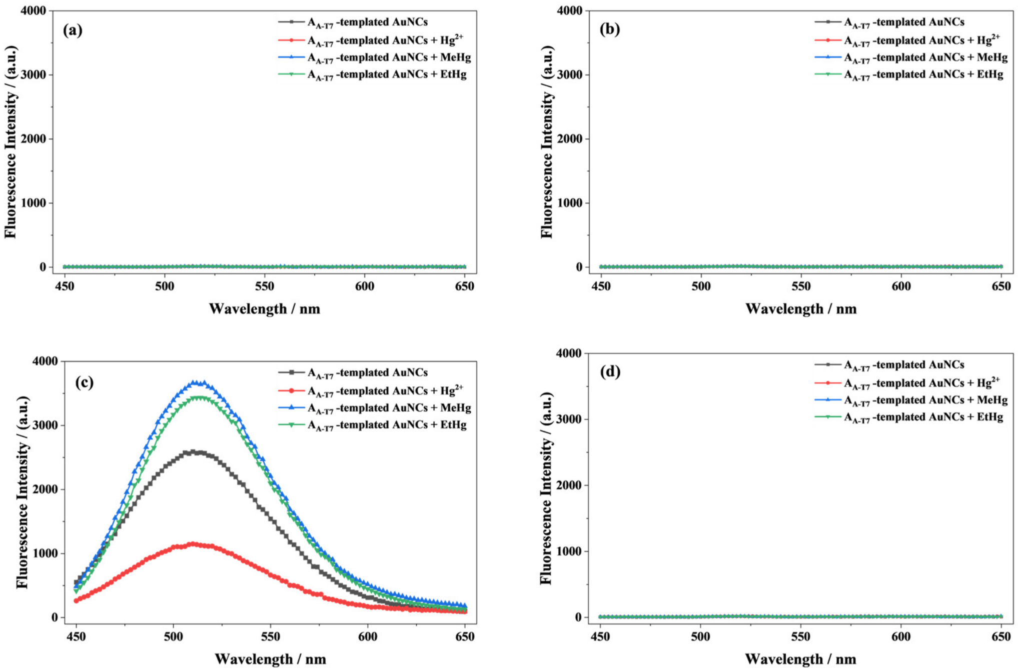

 https://doi.org/10.3390/bios16040218

Figure S7. Optimization of the concentration of $NaBH_4$, ranging from 0.2 mM to 0.8 mM. (a) 0.2 mM; (b) 0.4 mM; (c) 0.6 mM; (d) 0.8 mM.

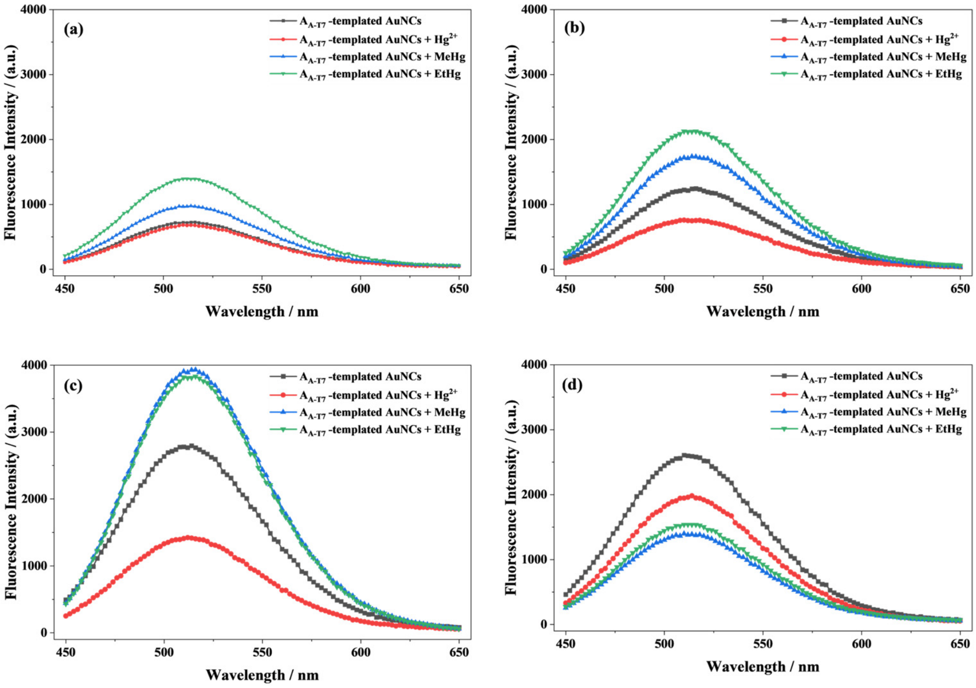

Figure S8. Optimization of pH in the system solution over the range of pH 6.5 to 8.0. (a) pH 6.5; (b) pH 7.0; (c) pH 7.5; (d) pH 8.0.

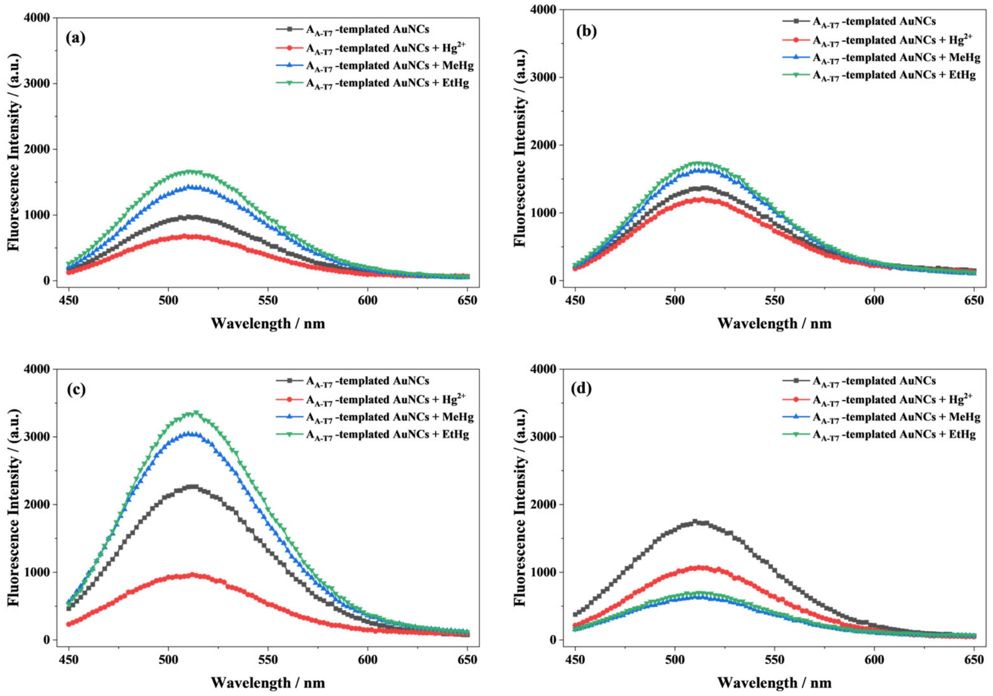

Figure S9. Optimization of concentrations of Au^{3+} ranging from 3.0 mM to 6.0 mM. (a) 3.0 mM; (b) 4.0 mM; (c) 5.0 mM; (d) 6.0 mM.

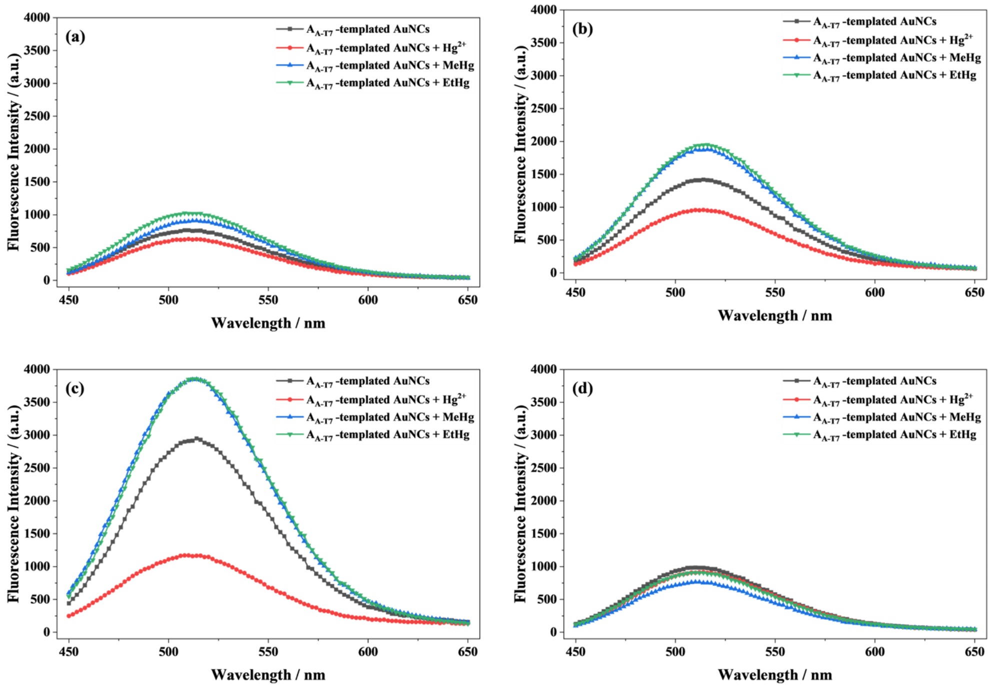

 https://doi.org/10.3390/bios16040218

Figure S10. Cell viability analysis of $A_{A\text{-}T7}$-templated AuNCs with/without Hg^{2+}, MeHg or EtHg (n=4).

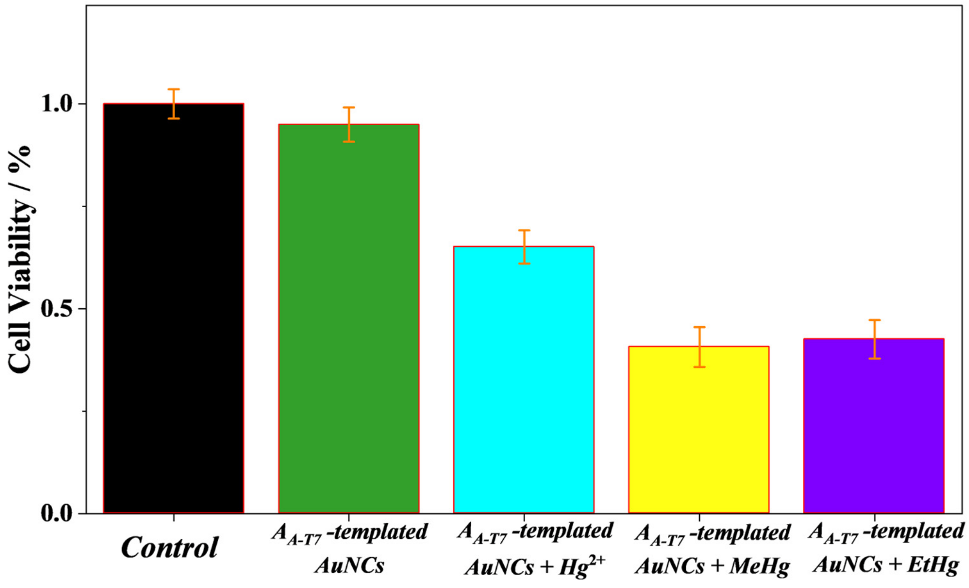

Figure S11. Intracellular confocal fluorescence imaging analysis. (a) HeLa cells at bright field; (b) HeLa cells at dark field; (c) HeLa cells incubated with $A_{A\text{-}T7}$-templated AuNCs at dark field; (d) HeLa cells co-incubated with $A_{A\text{-}T7}$-templated AuNCs and Hg^{2+} at dark field; (e) HeLa cells co-incubated with $A_{A\text{-}T7}$-templated AuNCs and MeHg at dark field; (f) HeLa cells co-incubated with $A_{A\text{-}T7}$-templated AuNCs and EtHg at dark field.

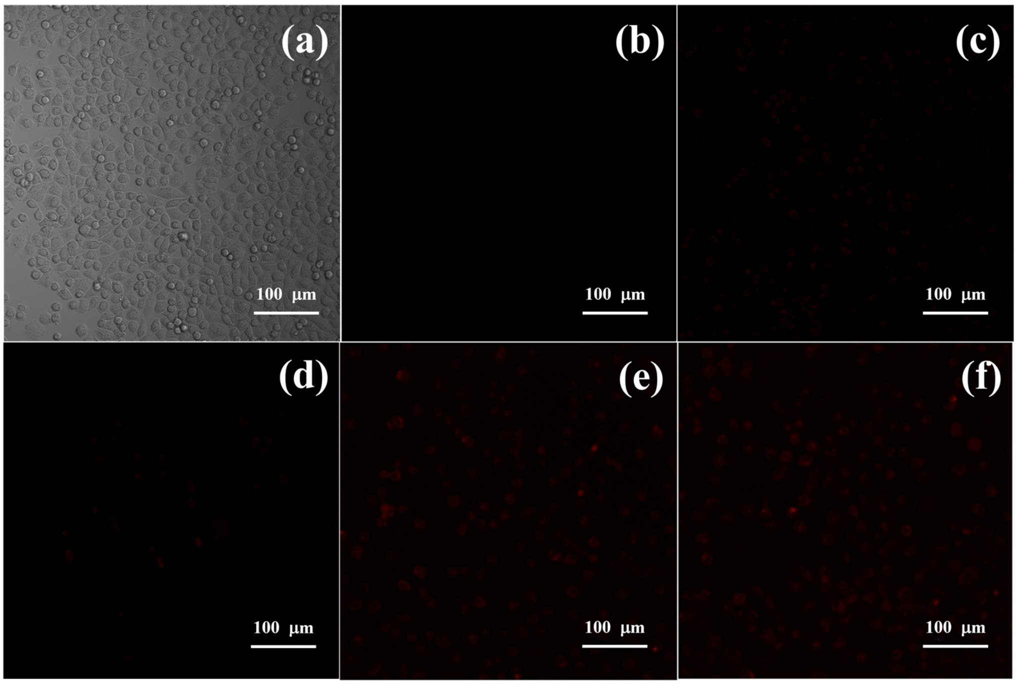

Supplement: Supplementary file 1 [file biosensors-16-00218-s001.zip › biosensors-4215509-supplementary.pdf]
